# Intracardiac echocardiography-guided transcatheter aortic valve implantation without contrast

**DOI:** 10.1093/ehjcr/ytae159

**Published:** 2024-03-28

**Authors:** Kanna Nakamura, Yasushi Fuku, Kazushige Kadota

**Affiliations:** Department of Cardiology, Kurashiki Central Hospital, 1-1-1 Miwa, Kurashiki 710-8602, Japan; Department of Cardiology, Kurashiki Central Hospital, 1-1-1 Miwa, Kurashiki 710-8602, Japan; Department of Cardiology, Kurashiki Central Hospital, 1-1-1 Miwa, Kurashiki 710-8602, Japan

**Figure:**
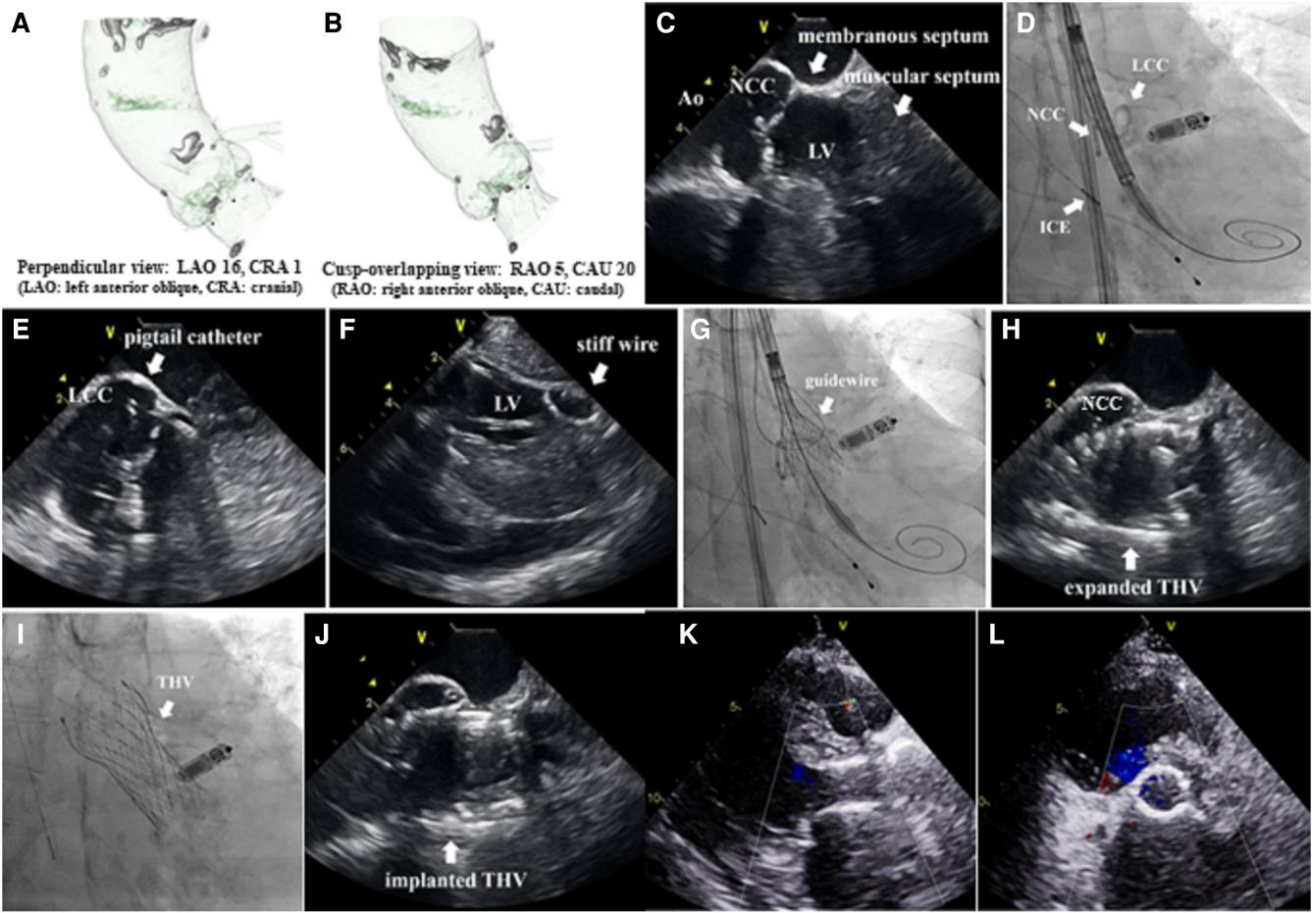


An 83-year-old woman with a leadless pacemaker implant for bradycardiac atrial fibrillation was admitted to the hospital for heart failure. She presented with severe renal dysfunction with a mean creatinine (3.92 mg/dL) and glomerular filtration rate (9.0 mL/min/1.73 m^2^). Transthoracic echocardiography (TTE) revealed severe aortic stenosis. We planned transcatheter aortic valve implantation using a transfemoral percutaneous approach with AcuNav (Biosense Webster, Irvine, CA, USA) intracardiac echocardiography (ICE) guidance under local anaesthesia. A contrast-enhanced computed tomography was performed after adequate rehydration for appropriate transcatheter heart valve (THV) size selection, perpendicular and the cusp-overlapping view (*Panels A* and *B*). Although 89 mL contrast was needed, it did not affect her renal function. We selected CoreValve Evolut Pro+® 26 mm (Medtronic Inc., Galway, Ireland). Intracardiac echocardiography inserted from the right internal jugular vein showed positional relationships between the non-coronary cusp (NCC), membranous, and muscular septum (*Panel C*; Supplementary material online, *Videos S1* and *S2*). Two pigtail catheters were attached to NCC and left coronary cusp (LCC) as landmarks, and the stiff wire was inserted. Intracardiac echocardiography showed the correct placement of wire in the left ventricle without the interference of the mitral valve chordae (*Panels D* and *F*; Supplementary material online, *Videos S3* and *S4*). The THV deployed using the cusp-overlapping view with a guidewire used as a landmark of the LCC, because the pigtail was removed. Intracardiac echocardiography visualized the appropriate depth of the implanted the THV (*Panel G* and *H*; Supplementary material online, *Video S5*). Intracardiac echocardiography and TTE after implantation showed no transvalvular and paravalvular leak and no aortic regurgitation (*Panels J–L*; Supplementary material online, *Videos S7* and *S8*). The mean aortic valve pressure gradient, measured by inserting a catheter into the left ventricle, improved to 10 mmHg (from 26 mmHg pre-implantation) after implantation. The procedure time was only 52 min and the fluoroscopy time was 19.7 min due to the advantages of ICE uninterrupted monitoring, no fluoroscopic interference, and high imaging resolution. Transthoracic echocardiography at discharge showed 1.28 cm^2^/m^2^ of aortic valve area index, no obvious aortic regurgitation with 1.8 m/s of peak aortic valve flow, and a mean aortic valve pressure gradient of 6 mmHg. She was discharged on the fifth post-operative day without worsening renal dysfunction and with no need for dialysis.

## Supplementary Material

ytae159_Supplementary_Data

## Data Availability

The data underlying this article are available in the article. No new data were generated or analysed in support of this research.

## Supplementary material

**Consent:** The authors confirm that written consent for submission and publication of this case report has been obtained from the patient in line with the COPE guidance, including the use of images and associated text.

**Funding:** None declared.

